# City-scale car traffic and parking density maps from Uber Movement travel time data

**DOI:** 10.1038/s41597-019-0159-6

**Published:** 2019-08-21

**Authors:** Arsam Aryandoust, Oscar van Vliet, Anthony Patt

**Affiliations:** Climate Policy Research Group, Environmental Systems Science Department, Swiss Federal Institute of Technology (ETH Zurich), Zurich, Switzerland

**Keywords:** Civil engineering, Environmental impact, Energy modelling

## Abstract

Car parking is of central importance to congestion on roads and the urban planning process of optimizing road networks, pricing parking lots and planning land use. The efficient placement, sizing and grid connection of charging stations for electric cars makes it even more important to know the spatio-temporal distribution of car parking densities on the scale of entire cities. Here, we generate car parking density maps using travel time measurements only. We formulate a Hidden Markov Model that contains non-linear functional relationships between the changing average travel times among the zones of a city and both the traffic activity and flow direction probabilities of cars. We then sample the traffic flow for 1,000 cars per city zone for each city from these probability distributions and normalize the resulting spatial parking distribution of cars in each time step. Our results cover the years 2015–2018 for 34 cities worldwide. We validate the model for Melbourne and reach about 90% accuracy for parking densities and over 93% for circadian rhythms of traffic activity.

## Introduction

Car parking is of central importance to congestion on roads and their social, environmental and economic impacts on society. An imbalance between on- and off-street parking prices for instance leads to cruising for cheaper on-street parking lots which in turn is responsible for 8–74% of traffic in downtown areas^1^. If we consider that cars that burn fuels other than electricity are directly responsible for 14% of global greenhouse gas emissions^2^ and 3.3 million premature annual deaths worldwide^3^, we find that there exist large potentials for environmental savings. Knowing where cars are parked at what times could support urban planning in the process of optimizing road networks, pricing parking lots and planning land use.

The electrification of the mobility sector makes it even more important to know where cars are parked at what times. At times and locations where large numbers of cars are parked with high density and must charge simultaneously for their upcoming trips, their additional electricity consumption can cause stresses to the local grid^4^. On the other side, the batteries of parked electric cars can be valuable storage capacities that can be used for balancing grid operation with large shares of intermittent renewable energy sources^5–7^. For an efficient placement, sizing and grid connection of charging stations, it is therefore important to know the spatio-temporal distribution of car parking densities on the scale of entire cities^8–10^.

The existing literature on urban traffic does not provide data on spatio-temporal parking densities on the scale of entire cities. Classic traffic system research aims at the development of optimal transport networks by minimizing congestion on roads^11,12^. The existing theories focus on the stream variables speed, flow and concentration of vehicles^13–16^; in these, density always refers to the concentration of vehicles on roads. Car parking density maps instead would require data about the number of cars parked in each region of a city at various times of a day. We find that the main barriers for measuring such data directly are the costs and efforts of placing and operating sensors.

In this analysis, we explore if car parking density maps on the scale of entire cities can be estimated from travel time measurements among different zones of a city only. We formulate a Hidden Markov Model in which states are the locations of cars and emission measurements are the changing travel times among the zones of that city throughout a day. We apply the model to travel time data that is measured from undertaken Uber rides^17^ and generate the desired parking density maps for 34 cities around the globe. We further provide complete code and instructions on extending the presented model for a wider range of traffic and parking system analyses^18^.

## Results

We generate car parking density maps from Uber travel time data for 34 cities worldwide^19^. We formulate non-linear functional relationships between the changing average travel times among the zones of a city and both the traffic activity and flow direction probabilities of cars. We derive origin-destination matrices for each hour of the day and for all zones of an entire town with these functional relationships. We then sample the traffic flow for 1,000 cars per city zone for each city from the resulting probability distributions and normalize the resulting spatial distribution of cars in each time step. The resulting parking density maps are hence independent from the number of sampled cars and can be scaled to arbitrary vehicle fleet sizes.

The resolution of the generated maps in time is one hour. The time periods for which we generate the results depend on the availability of the underlying Uber travel time measurements. At the time of writing this, Uber provides travel time data for the years 2015–2018 and distinguishes these by weekdays, weekends and the quarter of a year. In addition to these, Uber also provides travel time statistics that are collected regardless of the day type; these datasets cover a larger variety of trips than the separated datasets and therefore have lower sparsity.

**Fig. 1:**
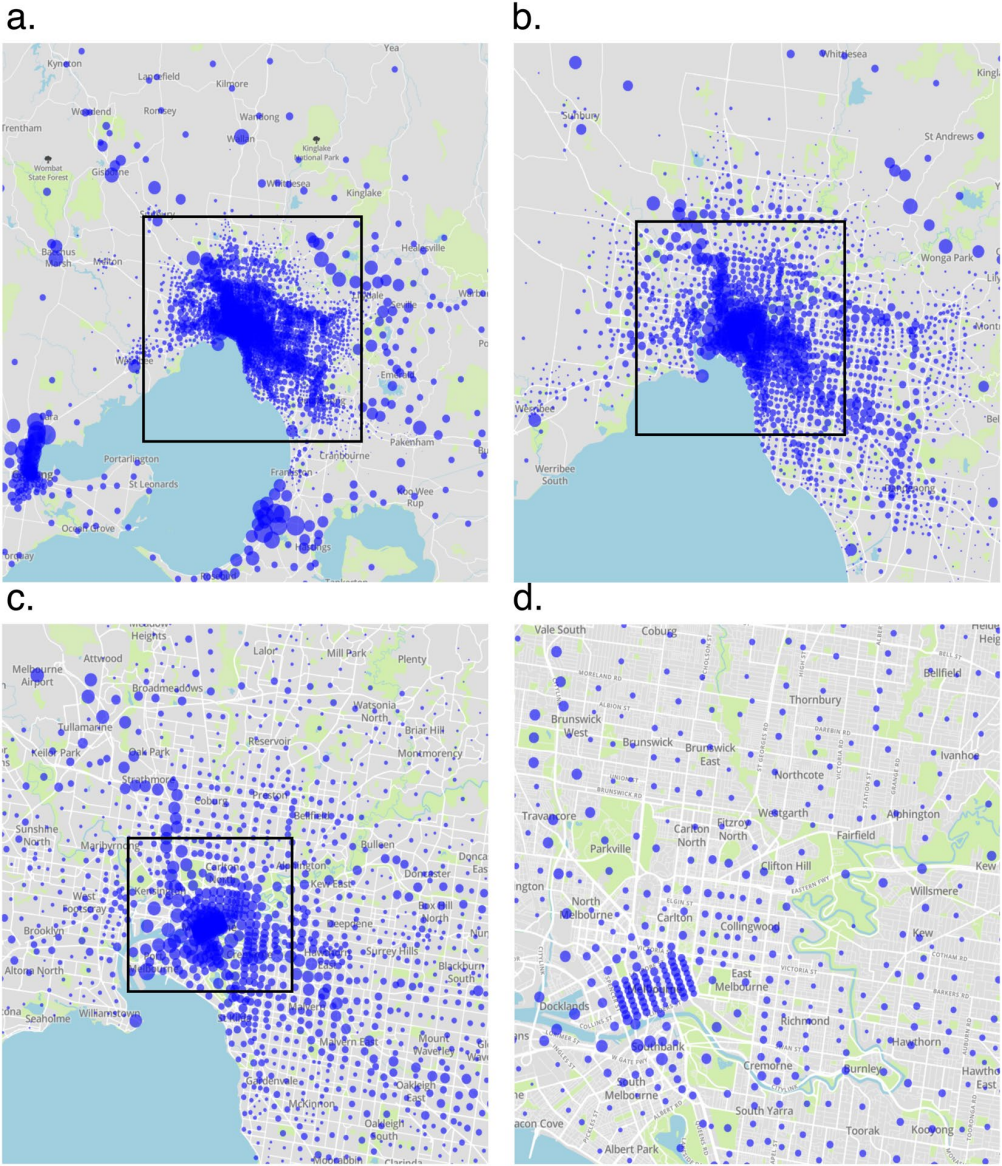
A scatter plot of the generated car parking densities for Melbourne on weekdays of the third quarter of 2017 (9:00 am) that shows the different spatial scales at which the results can be analyzed. The size of a blue dot indicates the density of parked cars in the respective city zone. The zoom factor increases within the marked black windows (zoom windows) from (**a**) to (**d**).

The resolution of the generated maps in space varies and depends on how Uber divides cities into different zones. Figure 1 shows the different scales at which the generated parking maps can be used. The parking maps can be used for an analysis of the entire suburban area of a town (Fig. 1a,b) or the center of a town (Fig. 1c); the accuracy is also adequate for an analysis of parking densities within the city center of a town (Fig. 1d).

We subsequently validate the presented results in two steps for the city of Melbourne during the years 2015–2017 for both travel time data that is separated by day types and those that are not separated by day types. In a first step, we validate the circadian rhythm of traffic activity that we generate by our model. In a second step, we validate the car parking densities that result from our traffic flow model for 100–105 city zones; the city zones that we validate are given by the location of the operated underground parking sensors in Melbourne. Our choice of Melbourne for validation is arbitrary and motivated by the availability of measured traffic count and parking density data; we make this choice independent from the performance of our model.

### Validation of traffic activities

A first substantial hypothesis in our model is, that whether a car drives to another city zone or stays parked in its origin zone is a function of changing travel times from the car’s current location to all possible destination zones throughout a day. This means, that the higher the measured travel times at a certain time of a day in a particular city zone are, the more likely it is that a car will undertake a trip to another destination at that given time of the day. This generates a characteristic circadian rhythm of traffic activity for each sampled day. We validate our sampled circadian rhythms with vehicle count data from street segments in the city of Melbourne that we use as an indicator for traffic activity^20^.

Table 1 contains the numeric results of the validation for the years 2015 until 2017. The percentual fit between the modeled and the measured circadian rhythm is 94–99% for weekdays and 93-99% for weekends. We observe that the fit is higher for weekdays when using mean travel time data that is not separated by day types; these datasets have usually lower sparsity than those that are separated by day types. Except for the last three quarters of the year 2016, the percentual validation fit is larger for weekends if one uses the travel time data that is separated by day types than when using non-separated data.

**Table 1:** Validation of traffic activity.

Year	Quarter	Weekday Fit	Weekend Fit
**Uber travel time data not separated by day type**			
2015	1^*st*^	97%	94%
	2^*nd*^	98%	93%
	3^*rd*^	98%	95%
	4^*th*^	98%	95%
2016	1^*st*^	99%	95%
	2^*nd*^	99%	99%
	3^*rd*^	99%	99%
	4^*th*^	99%	99%
2017	1^*st*^	99%	95%
	2^*nd*^	97%	95%
**Uber travel time data separated by day type**			
2015	1^*st*^	94%	94%
	2^*nd*^	96%	94%
	3^*rd*^	96%	98%
	4^*th*^	99%	98%
2016	1^*st*^	99%	98%
	2^*nd*^	99%	96%
	3^*rd*^	99%	96%
	4^*th*^	99%	96%
2017	1^*st*^	98%	99%
	2^*nd*^	97%	96%

The percentual fit between measured and modeled traffic activities using their Mean Squared Error as validation metric. The measured values are validated twice, once for the Uber travel time data that is measured during both day types (upper part), and once for the Uber travel time data that is separated by weekdays and weekends (lower part).

**Fig. 2:**
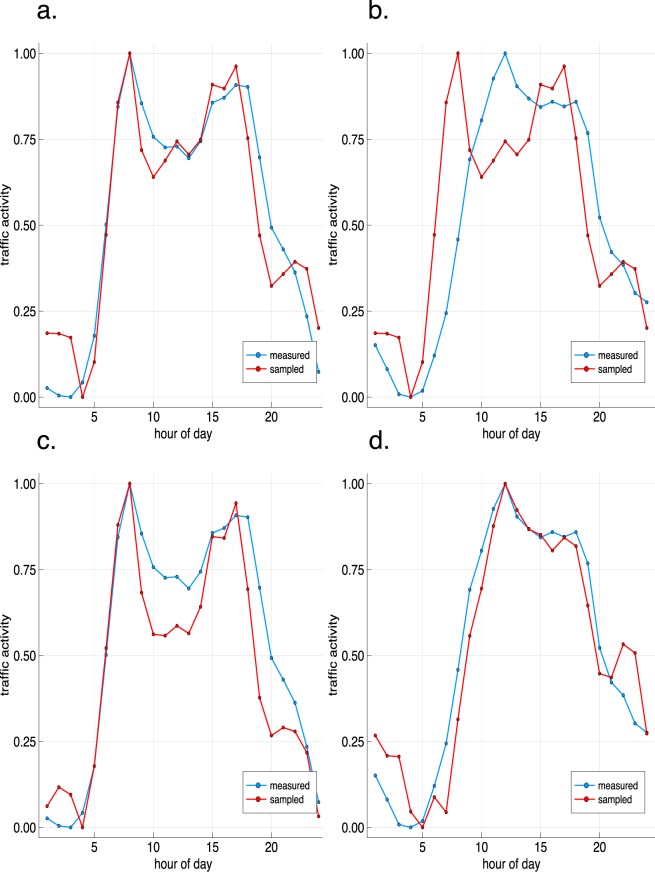
The measured (blue) and sampled (red) circadian rhythms of traffic activity for different datasets of Melbourne in the first quarter of the year 2017. (**a**) Weekdays with travel time data that is not separated by day type and a fit of 99%. (**b**) Weekends with travel time data that is not separated by day type and a fit of 95%. (**c**) Weekdays with travel time data that is separated by day type and a fit of 98%. (**d**) Weekends with travel time data that is separated by day type and a fit of 99%.

Figure 2 visualizes the results exemplarily for the first quarter of the year 2017. We can see that our sampled traffic activity is shifted towards the weekday patterns when using travel time data that is not separated by day type and therefore deviates from the actually measured traffic patterns when validated for measurements on weekends (Fig. 2b). For all other datasets (Fig. 2a,c,d), the sampled and measured traffic activities match with very high accuracy. The circadian rhythm of urban traffic has four characteristic features that are similar for all larger cities around the world^21–25^: first, a relatively high morning rush hour traffic between 8:00–10:00 am; second, a moderate lunch time traffic between 11:00 am–1:00 pm; third, a peak evening rush hour traffic between 4:00–6:00 pm; fourth, a relatively low midnight traffic between 1:00–3:00 am. We can observe that both the measured and the sampled circadian rhythms contain the four characteristic features of urban traffic activity.

### Validation of parking densities

The second substantial hypothesis in our model is that the destination zone that a car will choose for a trip is a function of the traffic activity in that zone at that given time of the day. This means, that the higher the measured travel times to a particular city zone are, the more likely it is that cars will choose that zone as a destination. This generates characteristic flow directions of cars and, together with the first hypothesis, the parking density of cars among the zones of a city. We validate the characteristics of our modeled car parking densities with underground parking sensor measurements in about 100 from the total of 2,357 modeled city zones in Melbourne^20^. The validated city zones are given by the location of the operated underground sensors which is mostly around the city center of Melbourne.

Table 2 contains the numeric results of the validation for the years 2015 until 2017. The percentual fit between sampled and measured parking densities is in a similar range for weekdays and weekends. We observe average fits of 82–92% for weekdays and 82–90% for weekends. The minimum fit is 64–83% for weekdays and 53–82% for weekends. The maximum fit is 94–97% for weekdays and 95–99% for weekends. The fit between the sampled and measured parking densities during weekdays is higher for the travel time data that is not separated by day type than for the separated data from 2015 until the first quarter of 2016. For the following time periods, the percentual fit is higher for the separated travel time datasets than for those that do not separate the data by day type. This turning point aligns with a larger decrease in the sparsity of the underlying travel time data for Melbourne from the first to the second quarter of 2016 (Table 3). During weekends, the percentual fit is always larger or equal for the non-separated datasets compared to the separated ones.

**Table 2:** Validation of parking densities.

Year	Quarter	Weekday Fit			Weekend Fit		
		Min	Max	Mean	Min	Max	Mean
**Uber travel time data not separated by day type**							
2015	1^*st*^	68%	96%	83%	70%	95%	85%
	2^*nd*^	75%	96%	86%	76%	95%	88%
	3^*rd*^	76%	94%	87%	80%	95%	88%
	4^*th*^	80%	95%	90%	57%	95%	90%
2016	1^*st*^	79%	95%	90%	80%	95%	90%
	2^*nd*^	78%	95%	90%	78%	95%	90%
	3^*rd*^	73%	95%	88%	73%	96%	88%
	4^*th*^	79%	97%	90%	81%	98%	90%
2017	1^*st*^	74%	98%	88%	76%	98%	90%
	2^*nd*^	73%	95%	88%	71%	96%	90%
	3^*rd*^	74%	95%	87%	71%	96%	88%
	4^*th*^	73%	95%	90%	77%	96%	90%
**Uber travel time data separated by day type**							
2015	1^*st*^	67%	94%	82%	53%	96%	82%
	2^*nd*^	71%	94%	85%	55%	96%	83%
	3^*rd*^	69%	94%	84%	75%	96%	85%
	4^*th*^	81%	96%	90%	63%	97%	87%
2016	1^*st*^	64%	95%	88%	80%	98%	87%
	2^*nd*^	75%	97%	90%	78%	97%	87%
	3^*rd*^	74%	95%	90%	75%	98%	85%
	4^*th*^	83%	96%	92%	77%	96%	87%
2017	1^*st*^	78%	94%	90%	80%	99%	88%
	2^*nd*^	76%	96%	90%	82%	97%	90%
	3^*rd*^	76%	95%	90%	71%	97%	87%
	4^*th*^	79%	95%	91%	78%	97%	88%

The columns Min fit, Max fit and Mean fit describe the minimum, maximum and average fits between sampled and measured parking densities in percentage when using the Mean Squared Error as validation metric.

**Table 3:** The sparsity of the currently provided Uber travel time datasets in percentage. Cities starting with A – M. The variable N indicates the total number of city zone polygons in which the respective city is divided.

		2015				2016				2017				2018			
		Q1	Q2	Q3	Q4	Q1	Q2	Q3	Q4	Q1	Q2	Q3	Q4	Q1	Q2	Q3	Q4
Amsterdam N = 181	All	—	—	—	—	82	75	73	72	71	66	64	64	64	60	58	59
	WD	—	—	—	—	85	79	78	77	76	72	70	70	70	66	65	65
	WE	—	—	—	—	88	83	81	79	79	74	72	72	73	69	67	68
Bangalore N = 198	All	—	—	—	—	24	19	9	9	12	12	13	12	12	10	11	12
	WD	—	—	—	—	30	24	12	13	17	16	17	17	16	14	15	16
	WE	—	—	—	—	39	33	19	19	24	22	24	24	24	21	22	24
Bogota N = 1,160	All	—	—	—	—	87	84	82	74	73	71	69	67	69	68	68	69
	WD	—	—	—	—	89	87	85	78	77	75	74	72	73	72	73	73
	WE	—	—	—	—	92	90	88	82	81	78	76	75	76	75	75	77
Boston N = 1,247	All	—	—	—	—	93	92	91	90	91	90	89	89	89	88	88	88
	WD	—	—	—	—	94	93	92	92	92	91	91	91	91	90	90	90
	WE	—	—	—	—	95	95	94	93	94	93	93	92	93	92	92	92
Brisbane N = 671	All	96	95	94	92	92	91	89	87	88	87	86	86	87	87	86	86
	WD	97	96	95	94	93	93	92	90	90	89	89	88	89	89	89	88
	WE	97	96	95	94	94	93	92	91	91	90	89	89	91	90	90	90
Brussels N = 724	All	—	—	—	—	96	94	93	91	90	88	87	86	85	84	84	82
	WD	—	—	—	—	97	95	95	93	92	91	90	89	88	87	87	85
	WE	—	—	—	—	98	97	97	95	94	94	93	92	92	91	91	90
Cairo N = 784	All	—	—	—	—	93	89	86	85	84	83	82	82	82	81	80	80
	WD	—	—	—	—	94	90	87	86	85	84	83	84	83	82	81	82
	WE	—	—	—	—	96	93	91	90	89	88	87	88	88	87	86	86
Cincinnati N = 454	All	—	—	—	—	90	88	86	85	84	82	82	81	81	81	80	80
	WD	—	—	—	—	92	91	89	88	87	86	85	85	84	84	84	84
	WE	—	—	—	—	94	93	92	91	91	89	88	88	88	88	88	88
Hyderabad N = 145	All	—	—	—	—	29	23	14	11	15	14	14	14	14	11	12	13
	WD	—	—	—	—	35	28	17	14	19	18	18	18	18	15	16	16
	WE	—	—	—	—	43	37	25	21	27	25	25	25	25	21	22	24
Johannesburg & Pretoria N = 934	All	—	—	—	—	97	96	95	95	95	95	94	94	94	94	94	94
	WD	—	—	—	—	97	97	96	96	96	95	95	95	95	95	95	95
	WE	—	—	—	—	98	97	97	97	97	97	96	96	97	96	96	96
Leeds N = 229	All	—	—	—	—	94	93	92	90	90	89	88	87	87	87	86	85
	WD	—	—	—	—	95	95	94	93	93	91	91	90	90	90	90	88
	WE	—	—	—	—	96	96	95	93	94	93	91	91	92	91	90	90
London N = 983	All	—	—	—	—	73	71	68	66	67	65	64	63	65	63	61	62
	WD	—	—	—	—	79	77	75	73	74	72	71	70	71	70	68	68
	WE	—	—	—	—	80	78	76	74	74	73	72	71	73	72	70	71
Los Angeles N = 2,716	All	—	—	—	—	89	87	85	84	84	84	83	83	82	82	82	82
	WD	—	—	—	—	91	90	87	87	87	87	86	85	85	85	85	85
	WE	—	—	—	—	93	93	91	90	90	90	89	89	89	89	89	89
Manchester N = 246	All	—	—	—	—	82	80	78	73	72	69	66	65	66	66	65	63
	WD	—	—	—	—	86	85	83	78	78	75	73	72	72	72	72	70
	WE	—	—	—	—	88	86	85	80	80	78	75	74	76	75	74	73
Melbourne N = 2,357	All	98	97	96	94	94	92	91	89	88	88	87	86	87	87	87	87
	WD	99	98	97	96	98	95	93	91	91	91	91	89	90	91	91	89
	WE	99	98	97	96	96	95	94	92	92	92	91	90	91	91	91	91
Miami N = 1206	All	—	—	—	—	84	82	81	80	78	78	79	77	76	79	79	78
	WD	—	—	—	—	86	85	84	83	81	81	82	80	79	82	82	81
	WE	—	—	—	—	90	89	88	87	86	86	87	85	85	86	87	86
Mumbai N = 695	All	—	—	—	—	75	72	67	60	59	57	58	55	55	53	53	53
	WD	—	—	—	—	78	75	70	64	63	61	62	59	59	57	58	57
	WE	—	—	—	—	82	79	75	69	69	67	67	65	65	63	63	63

**Fig. 3:**
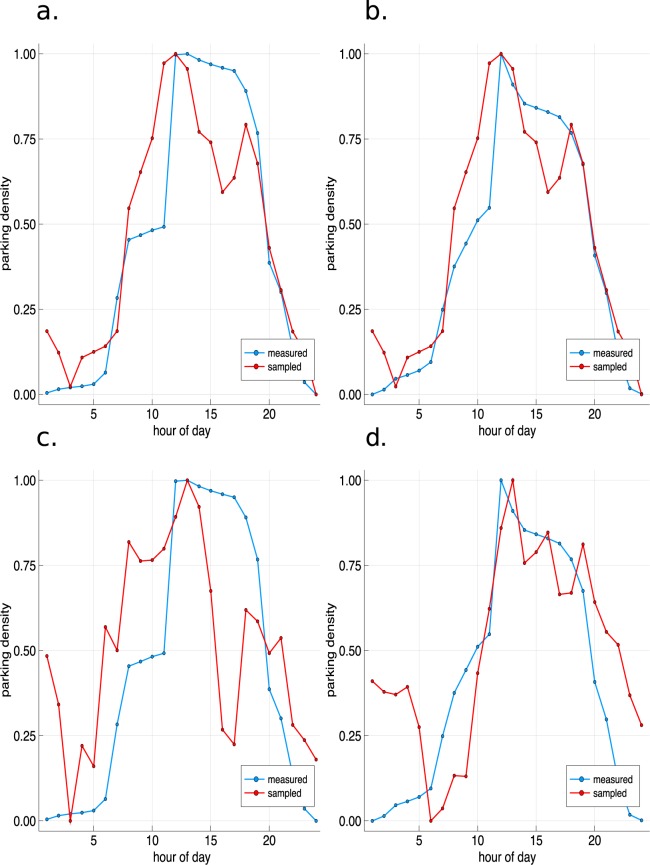
The measured (blue) and sampled (red) parking densities for different datasets of Melbourne in the exemplar city zone 956 in the fourth quarter of the year 2016. (**a)** Weekdays with travel time data that is not separated by day type and fit of 97%. (**b)** Weekends with travel time data that is not separated by day type and fit of 94%. (**c)** Weekdays with travel time data that is separated by day type and fit of 90%. (**d)** Weekends with travel time data that is separated by day type and fit of 94%.

Figure 3 further visualizes the validation results for an exemplar city zone during the fourth quarter of the year 2016 for both the non-separated (Fig. 3a,b) and the separated (Fig. 3c,d) datasets. We can observe that the patterns of parking density better match with the non-separated travel time data for both weekdays and weekends (Fig. 3a,b) than with the separated ones (Fig. 3c,d). In these, both the sampled (red) and measured (blue) parking densities rise, reach their peaks and decline at the same time. With the datasets that are separated by day types (Fig. 3c,d), our modeled (red) rise, peak and decline phases of parking density mismatch with the measured (blue) values.

## Discussion

We explore, if temporal car parking density maps can be modeled on the scale of an entire city, given travel time measurements between the zones of that city only. Using the Hidden Markov traffic Model that we present and the travel time data that is published by Uber, we find that parking densities can be estimated with about 90% accuracy for both weekdays and weekends. The circadian rhythm of daily commuting traffic can further be sampled with an accuracy of over 93% with our model. Although Uber users may not take an Uber ride for their daily commute but rather for extraordinary trips, which creates a large sparsity and bias in most of the data, we find that the measured travel time data is most of the time representative enough for creating the desired car parking density maps on the scale of entire cities.

The research question we ask is new and the data that we generate is the first of its kind in the literature. In contrast to classic traffic system research, our theory estimates the density of car parking by sampling individual driving behaviour. It contains elements of the Cumulative Vehicle Count Curves (N-curves)^26^ and Wardrop’s second principle of equilibrium^27^. Our theory, however, differs in the fundamental hypothesis that the density of cars parked in an area is a function of the changes in travel time between the origin and destination pairs that relate to this area throughout a day.

Our results are consistent with those of previously performed traffic system analyses^21–25^. The parking densities and commuting trends that we model based on our central hypotheses mostly match with those that are measured in the city of Melbourne^20^. We choose Melbourne for the validation of our results based on the parking and traffic data that is publicly available.

The performance of the presented model depends on the sparsity of the origin-destination travel time matrices that we use to sample the traffic flow of cars. We can observe that the travel time data that is collected without separation by weekdays and weekends has a lower sparsity and leads to more accurate parking density trends (Fig. 3a,b) than the travel time data that is separated by weekdays and weekends (Fig. 3c,d). For the modeled circadian rhythms of traffic activity, however, the opposite holds: the traffic activity features at weekends are better modeled with the travel time data that is separated by day types (Fig. 2d) than when using the non-separated data (Fig. 2b). One major reason is that the travel time statistics for the five weekdays have greater weight in the non-separated datasets than the two weekend days. However, this does not hold for the datasets of the year 2016 which could again be caused by biases that are given through the sparsity of the datasets. City zones can further behave as ever growing sinks if more cars flow into these than out of these; this is again caused by the sparsity in the underlying data. Further research can be done on reducing the sparsity of the underlying travel time data by using e.g. satellite imagery to estimate the missing travel time matrix entries of the Uber travel time data. This could reduce the ever growing sink characteristics of zones and further biases in the traffic flow that are given by the Uber data.

**Fig. 4:**
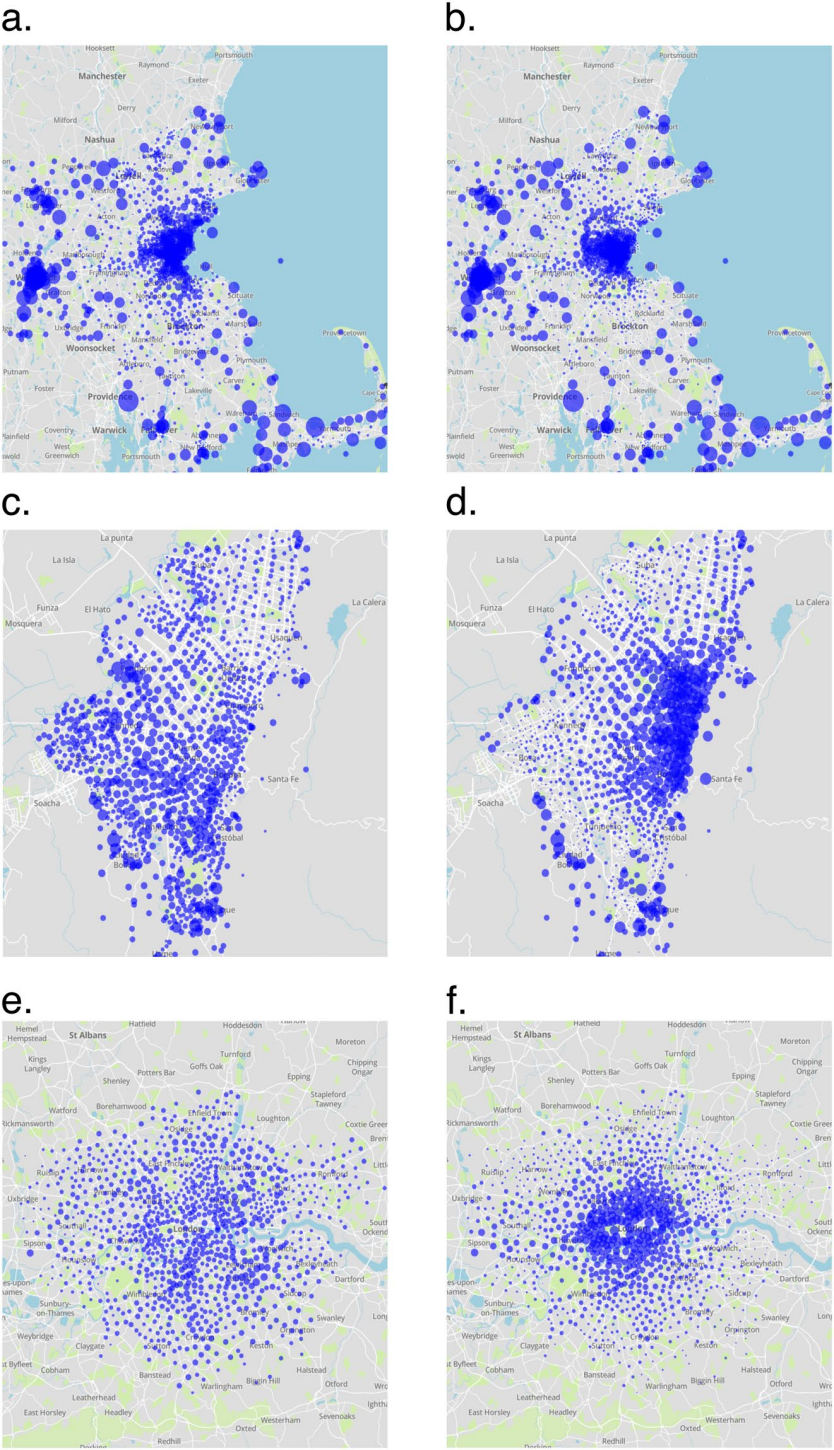
The parking density maps of Boston, Bogota and London at the times of their largest diversities. Each dot represents a city zone and the size of each dot represents the number of cars parked in the respective city zone at the given time. The maps on the left column represent parking at 4:00 am (**a,c,e**). The maps on the right column represent parking at 9:00 am (**b,d,f**).

Figure 4 visualizes the generated parking density maps for three more cities at the times of their largest diversity. The diversity of the visualized parking maps implies the importance of our generated data for the electrification of the mobility sector: policies that are found to be effective for charging electric cars in one city can be useless in other cities due to different patterns of car parking. On the other side, our data confirms that also generally applicable policies exist: the commuting behaviour of car users creates high parking densities at commercial centers during working hours. This always includes the times of peak solar power generation during midday at which grid balancing services of electric car batteries could be most useful.

## Methods

**Fig. 5:**
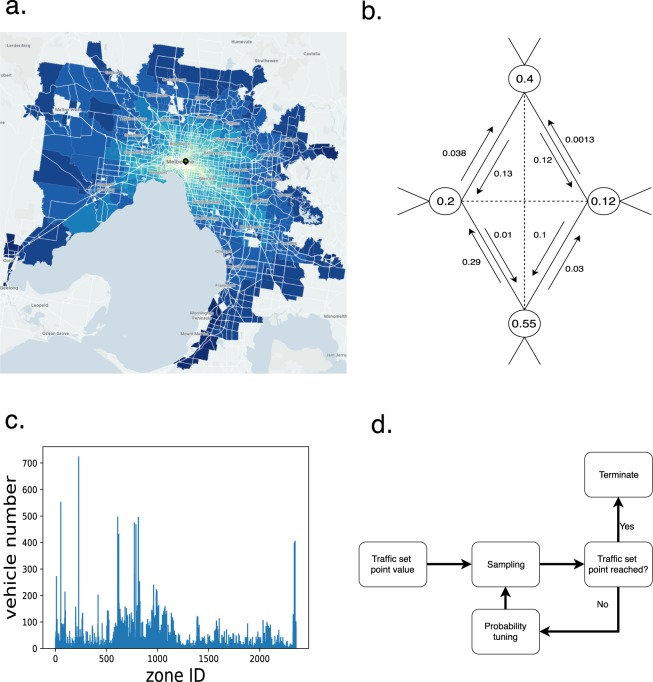
The core stages of the modeling method. (**a**) The city of interest (here e.g. Melbourne) is divided into zones for which we are given mean travel time data for a subset of these zones and day times. (**b**) A graph representation of the city zones. Each node stands for a zone and each value incorporated in the node stands for the probability that a car located in that zone drives. The probabilities of choosing a certain destination zone are assigned to each latency of the graph. The flow of cars between city zones is sampled from the joint probabilities of driving and choosing a destination zone. (**c**) The distribution of cars parked in each zone in each time step is calculated as a function of traffic flow between all zones. (**d**) A set of gradient descent algorithms performs model selection by tuning the open model parameters.

We are given the arithmetic mean of hourly travel time measurements between different zones of a city (Fig. 5a) and want to estimate the traffic flow and spatial parking distribution of cars in that city. In a first stage, we estimate the probabilities of car traffic between zones as a function of mean travel times (Fig. 5b). These probabilities exploit the changes in mean travel time between the zones of the city throughout a day to approximate information about when cars would drive and where they would drive to. In a second stage, we sample individual car traffic from these probability distributions and determine the number of cars that are parked in a zone as a function of cars flowing in and out from that zone (Fig. 5c). In a third and last stage, we use validation results to tune the parameters of the probability distributions that we sample from (Fig. 5d). For each model parameter or each set of model parameters that we can freely choose, a set point value is used to make a good choice. A set point value can for instance be the evaluation error between sampled and measured parking densities and traffic activity, or the average number of trips and travel distances.

### Uber travel time data

The Uber Movement project provides statistical data about travel times between different zones of a city. At the time of writing this, data is available for 34 cities worldwide with one second resolution. The data distinguishes between each quarter of several years. Depending on the city of interest, data is available from the years 2015 until 2018. For each quarter of these years, three types of datasets are available. The first type of dataset contains the aggregated measurements of travel time during weekdays and weekends. The second type of dataset contains measurements for weekdays only, and the third type of dataset contains measurements of weekends only. Detailed information on how the statistics were derived can be retrieved from Uber’s official methodology paper^28^. The data includes both central and suburban regions of a city which are divided into up to 5,260 zones. The raw travel data, as it is published by Uber, consists of four entry types. We let N be the number of zones in which the city is divided and T be the number of discrete time steps in which one day is divided. The four entry types of the travel data can then be defined for all t = 1…T and i, j = 1…N as:

- *μ*_*ij*,*t*_: = the mean travel time from zone i to zone j at daytime t
- *σ*_*ij*,*t*_: = the standard deviation of travel time from zone i to zone j at daytime t
- *geo*_*μ*_*ij*,*t*_: = the geometric mean travel time from zone i to zone j at daytime t
- *geo*_*σ*_*ij*,*t*_: = the geometric standard deviation of travel time from zone i to zone j at daytime t

### The sparsity of data

The sparsity of the datasets plays an important role for the performance of the here presented model. These sparsities depend on the number of Uber users in the provided cities and the provided time periods. Measurements are only given between zones and at times, in which a sufficient amount of Uber rides were undertaken, so as to ensure a sufficiently good representation of overall traffic^28^. This naturally generates a bias in the data that is larger the less Uber rides were undertaken in the time period and city of interest. Tables 3 and 4 give an overview of the sparsity of the currently provided datasets. We can see that the sparsity is generally lower for the datasets that contain travel time measurements for both day types. We further see that the sparsity of the datasets fluctuates but generally decreases over time as the user base of Uber grows and more rides are measured between city zones at various times of the day. We define the sparsity of a dataset as one minus the available number of data pairs divided by the maximum possible number of data pairs.

**Table 4:** Continuation of the sparsity of the currently provided Uber travel time datasets in percentage. Cities starting with N – W. The variable N indicates the total number of city zone polygons in which the respective city is divided.

		2015				2016				2017				2018			
		Q1	Q2	Q3	Q4	Q1	Q2	Q3	Q4	Q1	Q2	Q3	Q4	Q1	Q2	Q3	Q4
Nairobi N = 400	All	—	—	—	—	94	92	86	80	79	80	79	78	78	76	77	74
	WD	—	—	—	—	96	93	89	83	83	83	83	81	81	80	81	78
	WE	—	—	—	—	97	95	91	86	86	87	85	85	85	84	84	82
New Delhi N = 290	All	—	—	—	—	48	47	39	30	34	34	36	32	33	29	29	28
	WD	—	—	—	—	53	52	44	35	39	39	41	37	38	34	34	32
	WE	—	—	—	—	62	62	54	45	49	48	49	46	48	42	41	42
Orlando N = 1,893	All	—	—	—	—	94	94	93	92	91	91	92	90	91	92	92	91
	WD	—	—	—	—	95	95	94	94	93	93	93	92	93	93	94	93
	WE	—	—	—	—	97	97	97	96	96	96	96	95	96	96	96	96
Paris N = 5,260	All	—	—	—	—	96	95	94	93	93	92	92	92	95	92	93	93
	WD	—	—	—	—	96	96	95	94	94	94	94	94	95	93	94	94
	WE	—	—	—	—	97	97	97	96	96	96	95	95	95	95	96	96
Perth N = 173	All	—	—	—	—	73	70	67	62	63	63	62	58	60	62	60	57
	WD	—	—	—	—	79	77	74	69	70	70	69	66	67	68	67	64
	WE	—	—	—	—	80	77	75	70	72	71	69	67	69	70	68	67
Pittsburgh N = 608	All	—	—	—	—	93	91	91	90	90	89	89	88	88	88	86	86
	WD	—	—	—	—	94	93	93	92	92	91	91	90	90	90	89	89
	WE	—	—	—	—	96	95	94	94	94	93	93	92	93	92	91	92
San Francisco N = 2,710	All	—	—	—	—	96	96	95	95	95	95	94	94	94	94	94	94
	WD	—	—	—	—	97	96	96	96	96	95	95	95	95	95	94	94
	WE	—	—	—	—	98	97	97	97	97	97	96	96	96	96	96	96
Santiago N = 866	All	—	—	—	—	94	86	73	59	58	57	56	54	57	59	59	59
	WD	—	—	—	—	95	90	78	66	65	65	63	62	63	67	66	66
	WE	—	—	—	—	97	92	83	70	70	69	67	66	69	69	70	71
Sao Paulo N = 517	All	—	—	—	—	82	74	67	61	60	58	58	56	56	56	55	54
	WD	—	—	—	—	85	78	72	65	64	63	64	61	61	61	60	58
	WE	—	—	—	—	88	82	76	71	70	69	68	67	68	67	66	67
Seattle N = 776	All	—	—	—	—	94	93	92	91	91	91	90	90	90	90	89	90
	WD	—	—	—	—	95	94	93	93	93	92	92	92	92	91	91	91
	WE	—	—	—	—	96	95	95	94	94	94	93	93	94	93	93	93
Stockholm N = 776	All	—	—	—	—	96	93	94	93	93	92	92	91	92	91	90	90
	WD	—	—	—	—	95	95	96	95	95	94	94	94	94	93	93	92
	WE	—	—	—	—	95	95	97	96	96	95	95	94	95	94	94	94
Sydney N = 3,639	All	98	98	97	96	95	95	94	92	92	92	92	91	92	92	91	91
	WD	98	98	98	97	96	96	95	94	94	94	94	93	93	94	93	92
	WE	99	99	98	97	97	96	96	95	95	95	94	94	94	94	94	94
Taipei N = 691	All	—	—	—	—	76	73	70	67	78	84	78	75	74	71	70	69
	WD	—	—	—	—	79	77	74	71	81	86	81	79	77	75	74	73
	WE	—	—	—	—	85	83	80	77	88	92	86	84	83	81	79	79
Tampa Bay N = 503	All	—	—	—	—	82	81	80	78	75	75	77	75	75	77	77	77
	WD	—	—	—	—	85	84	83	82	80	80	81	79	79	81	81	80
	WE	—	—	—	—	89	88	87	85	84	84	85	83	83	85	85	85
Toronto N = 141	All	—	—	—	—	27	22	20	19	20	16	15	16	17	16	13	13
	WD	—	—	—	—	34	30	26	26	27	22	22	22	23	22	19	19
	WE	—	—	—	—	42	35	33	30	33	27	26	27	31	27	24	25
Washington D.C. N = 558	All	—	—	—	—	62	58	54	53	53	53	51	48	46	44	46	47
	WD	—	—	—	—	67	64	60	59	59	59	57	55	52	51	52	53
	WE	—	—	—	—	74	70	68	66	67	66	64	62	61	58	60	62
West Midlands UK N = 806	All	—	—	—	—	98	98	98	97	96	95	95	95	95	95	94	94
	WD	—	—	—	—	97	96	95	94	92	90	89	89	89	96	96	95
	WE	—	—	—	—	97	97	96	94	93	92	90	90	91	96	96	96

### City zones and their distances

Each zone of a city is described by a polygon with up to several hundred vertices. A separate file provides the latitudinal and longitudinal coordinates of these vertices for each polygon of each city. We let *S*_*i*_ be the number of vertices of the polygon of zone i, and *x*_*i*,*j*_ and *y*_*i*,*j*_ be the longitudinal and latitudinal coordinates of vertex j. We represent each city zone polygon by the centroid, also known as the center of gravity, of this polygon. We compute the area *A*_*i*_ of each city zone i and the coordinates of the i-th centroid (*long*_*i*_|*lat*_*i*_) for all i = 1…N and j = 1…*S*_*i*_, where j = 0 represents the same vertex as j = *S*_*i*_, as:

1 $${A}_{i}=\frac{1}{2}\cdot \mathop{\sum }\limits_{j=0}^{{S}_{i}}\,({x}_{i,j}\cdot {y}_{i,j+1}-{x}_{i,j+1}\cdot {y}_{i,j})$$

2 $$lon{g}_{i}=-\frac{1}{6{A}_{i}}\cdot \mathop{\sum }\limits_{j=0}^{{S}_{i}}\,({x}_{i,j}+{x}_{i,j+1})\cdot ({x}_{i,j}\cdot {y}_{i,j+1}-{x}_{i,j+1}\cdot {y}_{i,j})$$

3 $$la{t}_{i}=-\frac{1}{6{A}_{i}}\cdot \mathop{\sum }\limits_{j=0}^{{S}_{i}}\,({y}_{i,j}+{y}_{i,j+1})\cdot ({x}_{i,j}\cdot {y}_{i,j+1}-{x}_{i,j+1}\cdot {y}_{i,j})$$

The easiest way to calculate the distance between two points on the planet is to assume that latitude and longitude are straight lines. Using a constant distance of 111.3 km between each latitudinal degree and an average distance of 71.5 km per longitudinal degree, we can simply calculate the distances with the Pythagorean theorem. A more accurate way of doing this is to consider that longitudinal lines are not simply straight but rather bend depending on the latitudinal position: the distance between two longitudinal lines is around 111.3 km at the equator and 0 at the north and south poles. The dependence on the latitudinal position *lat* can then be expressed as 111.3 km ⋅ cos(lat). If we replace the average distance of 71.5 km per longitudinal degree with this expression within the Pythagorean theorem, we can calculate the distance *d*_*ij*_ between two zones i and j at the points (*long*_*i*_|*lat*_*i*_) and (*long*_*j*_|*lat*_*j*_) as:

4 $${d}_{ij}={d}_{ji}=111.3\,km\cdot \sqrt{co{s}^{2}\left(\frac{la{t}_{i}+la{t}_{j}}{2}\right)\cdot {(lon{g}_{i}-lon{g}_{j})}^{2}+{(la{t}_{i}-la{t}_{j})}^{2}}$$

### Hidden markov traffic Model

We define the state of the traffic system as the distribution of cars among city zones. The state in each time step belongs to a finite set of possible states if the number of sampled cars is finite and constant. We can extend the state space of our traffic model by assigning more properties to a car than just its location. Such properties can for instance be the states of charge of electric car batteries or the number of transported persons. We describe the location of a car at a given time t = 1…T by its city zone and introduce:

- *X*_*t*_: = the distribution of cars among all city zones at time step t

If we let V be the number of cars that we want to simulate, the state space Ω_*X*_ contains *N*^*V*^ possible arrangements. Every state *X*_*t*_ is only dependent on its previous state *X*_*t*−1_ which gives us a discrete-time Markov chain model. For the state transition probabilities at times t = 1…T − 1 it hence holds that:

5 $$P({X}_{t+1}| {X}_{t},{X}_{t-1},...,{X}_{1})=P({X}_{t+1}| {X}_{t})$$

We let the state transition of our model be given by the uncertain flow of cars among all city zones and denote this as ${\varepsilon }_{t}$. The number of possible state transitions is hence (*N*^*V*^)^2^. For each time step t = 1…T-1, we write:

6 $${X}_{t+1}={X}_{t}+{\varepsilon }_{t}$$

The transition probabilities can then be described by:

7 $$P({X}_{t+1}| {X}_{t})=P({X}_{t+1}-{X}_{t})=P({\varepsilon }_{t})$$

The problem that we want to solve can be formulated as finding the car flow probabilities $P({\varepsilon }_{t})$ given the mean travel times *μ*_*ij*,*t*_ only. This gives us a Hidden Markov Model as the number of cars flowing among city zones is not directly measured but derived as a function of the changing mean travel time throughout the day. The mean travel times are called emission measurements.

### Probabilities of driving and parking

For describing whether a car drives to another zone or stays parked in the same zone, we introduce a binary random variable:

- A: = binary random variable that describes whether a car drives or parks
  We denote the values that a random variable can assume in lower cased letters and let 0 stand for parking and 1 for driving, hence *a* ∈ Ω_*a*_ = {0, 1}. We determine the probabilities for possible values that A can take by letting the driving activity of cars in a zone be a function of the sum of mean travel times from that zone to all other zones. We assign a probability of driving according to the sum of mean travel time out of a zone in each hour compared to its minimum and maximum values throughout the entire day. For this purpose, we introduce two parameters:
- *p*_*min*_: = the probability that a car will drive, given the minimum sum of mean travel time out of a zone
- *p*_*max*_: = the probability that a car will drive, given the maximum sum of mean travel time out of a zone

One can assign arbitrary initial values to *p*_*min*_ and *p*_*max*_ and perform a model selection based on validation results to make a good choice of these parameters. We later present an algorithm that utilizes validation results of average driving times for parameter tuning. Two conditions that *p*_*min*_ and *p*_*max*_ must satisfy are:

8 $${p}_{max} > {p}_{min}$$

9 $${p}_{min},{p}_{max}\in [0,1]$$

For each zone of the city, we derive a set of 24 Binomial probability distributions, that is one for each hour of the day. We define the probabilities of an event P(A = a) for zones i = 1…N and times t = 1…T as:

10 $$P(A=1)={p}_{i,t}^{drive}=\left\{\begin{array}{ll}{p}_{min}+({p}_{max}-{p}_{min})\frac{{\sum }_{j=1}^{N}\,{\mu }_{ij,t}-mi{n}_{t}\{{\sum }_{j=1}^{N}\,{\mu }_{ij,t}\}}{ma{x}_{t}\left\{{\sum }_{j=1}^{N}\,{\mu }_{ij,t}\right\}-mi{n}_{t}\left\{{\sum }_{j=1}^{N}\,{\mu }_{ij,t}\right\}} & ma{x}_{t}\left\{\mathop{\sum }\limits_{j=1}^{N}\,{\mu }_{ij,t}\right\} > 0\\ 0 & ma{x}_{t}\left\{\mathop{\sum }\limits_{j=1}^{N}\,{\mu }_{ij,t}\right\}=0\end{array}\right.$$

11 $$P(A=0)=1-{p}_{i,t}^{drive}$$

The high sparsity of our available datasets can likely create a bias if data is missing more frequently at particular times and between particular zones of a city than at others. Considering the distance between the set of zones and the number of zones in each time step for which data points are available, can significantly decrease these biases. With *M*_*ij*,*t*_ being the subset of travel time data that is available at time step t, a formulation of Eq. (10) that is more robust against sparsity is given by:

12 $$P(A=1)={p}_{i,t}^{drive}=\left\{\begin{array}{ll}{p}_{min}+({p}_{max}-{p}_{min})\frac{1}{| {M}_{ij,t}| }\frac{{\sum }_{j\in {M}_{ij,t}}\,\frac{{\mu }_{ij,t}}{{d}_{ij}}-mi{n}_{t}\left\{{\sum }_{j\in {M}_{ij,t}}\,\frac{{\mu }_{ij,t}}{{d}_{ij}}\right\}}{ma{x}_{t}\left\{{\sum }_{j\in {M}_{ij,t}}\,\frac{{\mu }_{ij,t}}{{d}_{ij}}\right\}-mi{n}_{t}\left\{{\sum }_{j\in {M}_{ij,t}}\,\frac{{\mu }_{ij,t}}{{d}_{ij}}\right\}} & | {M}_{ij,t}|  > 0\\ 0 & | {M}_{ij,t}| =0\end{array}\right.$$

### Probabilities of choosing a destination

For describing which destination zone a car would choose for a trip, we introduce a second random variable:

- B: = discrete random variable that describes the destination zone that a car would choose

We also let the popularity of destination zones be a function of changing travel times throughout a day and ask ourselves how likely it is that a car in zone i travels to zone j at a given point t in time. For this purpose, we compare the mean travel time of an origin and destination pair in each hour with its minimum and maximum values throughout the entire day. We hence assign a numeric value between 0 and 1 to each origin and destination pair and for each direction. Note that we do not use the sums of mean travel times here but rather perform a min-max scaling of the data in each time step. The closer this value is to one, the more popular a destination zone is at that given hour. We generate a valid Multinomial probability distribution whose probabilities sum up to 1 for each zone and each hour of the day by normalizing all values with a factor *Z*_*i*,*t*_. The set of values that B can assume is given as *b* ∈ Ω_*b*_ = {1, ..., *N*}. We define the probability P(B = b) that a car in zone i = 1…N chooses a destination zone j = 1…N at time t = 1…T and their respective normalization factors as:

13 $$P(B=j)={p}_{ij,t}^{dest}=\left\{\begin{array}{ll}\frac{1}{{Z}_{i,t}}\frac{{\mu }_{ij,t}-mi{n}_{t}\{{\mu }_{ij,t}\}}{ma{x}_{t}\{{\mu }_{ij,t}\}-mi{n}_{t}\{{\mu }_{ij,t}\}} & ma{x}_{t}\{{\mu }_{ij,t}\} > 0\\ 0 & ma{x}_{t}\{{m}_{ij,t}\}=0\end{array}\right.$$

14 $${Z}_{i,t}=\mathop{\sum }\limits_{j=1}^{N}\,{p}_{ij,t}^{dest}$$

### Joint origin-destination probabilities

The desired transition probabilities $P({\varepsilon }_{t})$ of the traffic flow can now be described by the joint events of driving and choosing a destination. For a simplified notation of these joint probabilities, we introduce a third random variable:

- C: = discrete random variable that describes the joint events of driving (A = a) and choosing a destination (B = b)

We describe the state space of C with *c* ∈ Ω_*C*_ = {0, ..., *N*}. A value of zero means that a car does not drive (A = 0) and hence stays parked in its origin zone. Any other value indicates that a car is chosen to drive (A = 1) and chooses the respective destination zone (B = 1…N) that is given by the value of C other than zero. For a simplified notation and calculation, we assume the random variables A and B to be independent and write:

15 $$P(A=a,B=b)=P(A=a)\cdot P(B=b)$$

Given the independence of A and B, we calculate the probabilities of cars in zone i = 1…N to drive to a destination zone j = 1…N or stay parked at time t as:

16 $$P(C=0)=P(A=0)=(1-{p}_{i,t}^{drive})\cdot {\underbrace{\mathop{\sum }\limits_{j=1}^{N}{p}_{ij,t}^{dest}}}_{=1(normalized)}=(1-{p}_{i,t}^{drive})\quad (Marginalization)$$

17 $$P(C=j)=P(A=1,B=j)={p}_{i,t}^{drive}\cdot {p}_{ij,t}^{dest}$$

Note that the same holds for both the arithmetic and geometric mean of travel time. We summarize that the uncertain flow of cars among the zones of a city ${\varepsilon }_{t}$ is modeled as a function of mean travel times *μ*_*ij*,*t*_ and described by the probability distribution of *P*(*C* = *c*) as:

18 $$P({\varepsilon }_{t}) \sim {p}_{ij,t}^{joint}=\left\{\begin{array}{cc}1-{p}_{i,t}^{drive} & c=0\\ {p}_{i,t}^{drive}\cdot {p}_{ij,t}^{dest} & c > 0\end{array}\right.$$

### Initial value problem

To sample the traffic flow, we need to define a realistic initial state of the system. The problem of finding such a state is commonly referred to as the Initial Value Problem. We introduce:

- *X*_0_: = the initial distribution of cars among all city zones

We uniformly assign a location zone to each car and calculate the state transitions for an entire day. We expect the initial uniform distribution of cars to converge towards a naturally shaped distribution after all transition probabilities are applied to the traffic system. We then assume the last state of an entirely modeled traffic day as the solution to the Initial Value Problem and set:

19 $${X}_{0}\leftarrow {X}_{T}$$

### State transition properties

To analyze any values of interest, we can assign state transition properties to each undertaken trip. Here, we let the transition properties of our model be the travel distance and duration of each trip. Similar to the state of the system, also the state transition properties can be extended. Such extensions can for instance be carbon emissions, fuel consumption and transported amount of people for each trip. We introduce for all v = 1…V, i, j = 1…N and t = 1…T:

- ${T}_{v,ij,t}^{dur}$: = the duration of a trip undertaken by car v from zone i to zone j at time t
- ${T}_{v,ij,t}^{dis}$: = the distance of a trip undertaken by car v from zone i to zone j at time t

As for many other natural processes, we assume the stochastic distribution of travel duration and distance to be Gaussian^29^. We use the mean (*μ*_*ij*,*t*_) and standard deviation (*σ*_*ij*,*t*_) from the original datasets to describe the stochastic distributions of ${T}_{v,ij,t}^{dur}$; the calculated distances between city zones (*d*_*ij*_) are further used for describing ${T}_{v,ij,t}^{dis}$. We arbitrarily choose a standard deviation of 0.1 times the distance *d*_*ij*_ for creating a larger variety of individual trip distances. In order to avoid negative duration and distances, we truncate the probability distributions. We choose arbitrary lower and upper ranges of 0.1 times the mean and normalize the probability density functions accordingly. We let *t*^*dur*^ and *t*^*dis*^ describe random variables and formulate the truncated probability distributions of ${T}_{v,ij,t}^{dur}$ and ${T}_{v,ij,t}^{dis}$ as:

20 $${T}_{v,ij,t}^{dur} \sim \frac{\frac{1}{\sqrt{2\pi {\sigma }_{ij,t}^{2}}}\cdot exp-\frac{{({t}^{dur}-{\mu }_{ij,t})}^{2}}{2{\sigma }^{2}}}{\frac{1}{\sqrt{2\pi {\sigma }_{ij,t}^{2}}}\cdot {\int }_{-\infty }^{1.1{\mu }_{ij,t}}\,exp-\frac{{({t}^{dur}-{\mu }_{ij,t})}^{2}}{2{\sigma }^{2}}d{t}^{dur}-\frac{1}{\sqrt{2\pi {\sigma }_{ij,t}^{2}}}\cdot {\int }_{0.9{\mu }_{ij,t}}^{\infty }\,exp-\frac{{({t}^{dur}-{\mu }_{ij,t})}^{2}}{2{\sigma }^{2}}d{t}^{dur}}$$

21 $${T}_{v,ij,t}^{dis} \sim \frac{\frac{1}{\sqrt{2\pi {(0.1\cdot {d}_{ij})}^{2}}}\cdot exp-\frac{{({t}^{dis}-{d}_{ij})}^{2}}{2{(0.1\cdot {d}_{ij})}^{2}}}{\frac{1}{\sqrt{2\pi {(0.1\cdot {d}_{ij})}^{2}}}\cdot {\int }_{-\infty }^{1.1{d}_{ij}}\,exp-\frac{{({t}^{dis}-{d}_{ij})}^{2}}{2{(0.1\cdot {d}_{ij})}^{2}}d{t}^{dis}-\frac{1}{\sqrt{2\pi {(0.1\cdot {d}_{ij})}^{2}}}\cdot {\int }_{0.9{d}_{ij}}^{\infty }\,exp-\frac{{({t}^{dis}-{d}_{ij})}^{2}}{2{(0.1\cdot {d}_{ij})}^{2}}d{t}^{dis}}$$

### Sampling

We sample the state transitions and therewith the traffic of cars among all city zones by generating a random variable between 0 and 1 for each car in each time step. We then compare the value of the random variable with one of the Multinomial distributions of ${p}_{ij,t}^{joint}$ and decide for each car in which zone it stays parked or to which zone it drives. Which Multinomial distribution we choose from ${p}_{ij,t}^{joint}$ depends on the location of each car, that is i, and the time step of simulation which is t. The distance and duration of trips are sampled by generating random variables from the truncated Gaussian distributions in (20) and (21) for each trip.

### Model selection for *e*_*drive*_ and *e*_*dest*_

The parameters *e*_*drive*_ and *e*_*dest*_ determine the functional relationship between travel time data and the joint Origin-Destination probabilities of the modeled traffic system. In order to find a good choice for them, we must evaluate the values of interests in our model with real world observations. The values that we are mainly interested in in this modeling task are the circadian rhythm of traffic activity and the spatio-temporal distribution of car parking densities. We hence use measurements of these two values to make a good choice for *e*_*drive*_ and *e*_*dest*_. The subsequent subsections describe how the validation works in detail. In this subsection we describe an algorithm for finding a good solution for both parameters. Finding optimal values for *e*_*drive*_ and *e*_*dest*_ turns out to be a non-convex optimization problem. Optimizing via gradient descent is hence strongly dependent on the initial parameter values. The first part of the optimization process is hence about finding a good starting point. In order to do this, we evaluate the results of traffic activity and parking density for several values of *e*_*drive*_ and *e*_*dest*_ between zero and one, as well as between one and e.g. 10. Both *e*_*drive*_ and *e*_*dest*_ are used as exponents in the functional relationship of Eq. (18). We stretch the probability distributions for larger values than one and clinch these for smaller values than one, compared to a linear functional relationship that is given for a value of one for *e*_*drive*_ and *e*_*dest*_. We let n be the n-th iteration of the optimization algorithm and introduce:

- *e*^(*n*)^: = current parameter
- ${e}_{b}^{(n)}$: = best parameter until now
- ${\rm{\nabla }}{e}^{(n)}={e}^{(n)}-{e}_{b}^{(n)}$: = current parameter gradient
- *E*^(*n*)^: = evaluation error with current parameter
- ${E}_{b}^{(n)}$: = best evaluation error until now
- $\nabla {E}^{(n)}={E}^{(n)}-{E}_{b}^{(n)}$: = current error gradient
- *η*^(*n*)^ = *η*: = constant step size parameter

We test different initial parameters and set the one with the lowest evaluation error as the best found parameter ${e}_{b}^{(n)}$ with the best found evaluation error ${e}_{b}^{(n)}$ until now. In each iteration n, we calculate the evaluation error *E*^(*n*)^ that is given with the current parameter *e*^(*n*)^ as e.g. the Mean Squared Error between modeled and measured target values. The parameter *e*^(*n*)^ can thereby be either *e*_*drive*_ or *e*_*dest*_. Then, we calculate the new parameter gradient ∇*e*^(*n*)^ and the new error gradient ∇*E*^(*n*)^. We reset the current parameter value in the case that the current parameter does not improve the best evaluation error (∇*E*^(*n*)^ > 0):

22 $${e}^{(n)}\leftarrow {e}_{b}^{(n)}$$

In the case that the current parameter improves the best evaluation error until now (∇*E*^(*n*)^ < 0), we update the best found values:

23 $${e}_{b}^{(n)}\leftarrow {e}^{(n)}\wedge {E}_{b}^{(n)}\leftarrow {E}^{(n)}$$

We use the previous values for updating our model parameter towards a decreasing evaluation error for the next iteration:

24 $${e}^{(n+1)}={e}^{(n)}-\eta \cdot \frac{\nabla {E}^{(n)}}{\nabla {e}^{(n)}}$$

### Model selection for *p*_*min*_ and *p*_*max*_

The parameters *p*_*min*_ and *p*_*max*_ determine the range of probabilities for deciding whether a car drives or parks given the time of day and zone of presence; they were initially set to arbitrary values that satisfy the conditions in (8) and (9). Once we have first sampling results, we can use *p*_*min*_ and *p*_*max*_ to normalize our traffic system according to one or more set point values. We defined the two state transition properties travel duration and travel distance which we can use both for normalization. Here, we use the share of driving time within the lifetime of a vehicle for normalization and introduce:

- *A*_*set*_: = the realistic amount of driving time
- *A*_*drive*_: = the sampled amount of driving time

If we assume that the realistic amount of driving time within the lifetime of a car is around 5%, the set point value to which we must approach *A*_*drive*_ is *A*_*set*_ = 0.05. Alternatively, one can use other values such as the average number of trips, the average fuel consumption or the average travel distance of cars as values for normalization. The only condition is that these values must be recorded as state transition properties. A computationally effective way is to normalize each dataset separately. In this case, we normalize the traffic system with respect to the time steps t = 1…T and calculate:

25 $${A}_{drive}=\frac{{\sum }_{v=1}^{V}\,{\sum }_{t=1}^{T}\,{T}_{v,ij,t}^{dur}}{V\cdot {c}_{time}}$$

The constant *c*_*time*_ considers the time resolution of our simulation. For our analysis, the original datasets entail mean travel times *μ*_*ij*,*t*_ with one second resolution. This gives a time constant of *c*_*time*_ = 24⋅60⋅60. For one minute resolution, we would respectively calculate *c*_*time*_ = 24⋅60, and *c*_*time*_ = 24 for an hourly resolution. A more accurate but computationally intensive way is to normalize the traffic system throughout all datasets that are available for each city. In this case, we consider weekly, seasonal and inter annual variations of traffic patterns. With G being the number of datasets that we want to normalize and ${({T}_{v,ij,t}^{dur})}^{(g)}$ being the transition property of the g-th sample outcome, we calculate:

26 $${A}_{drive}=\frac{{\sum }_{g=1}^{G}\,{\sum }_{v=1}^{V}\,{\sum }_{t=1}^{T}\,{({T}_{v,ij,t}^{dur})}^{(g)}}{G\cdot V\cdot {c}_{time}}$$

We use a numeric iterative algorithm to iteratively calculate the parameter values that approach *A*_*drive*_ to *A*_*set*_. In each iteration, we update either *p*_*min*_ or *p*_*max*_ by solving one of two equations. Which parameter we update, depends on the relation between *A*_*drive*_ and *A*_*set*_. In each n-th iteration, we calculate:

27 $${p}_{max}^{(n+1)}={p}_{max}^{(n)}+({p}_{max}^{(n)}-{p}_{min}^{(n)})\frac{{A}_{set}^{(n)}-{A}_{drive}^{(n)}}{n\cdot {A}_{drive}^{(n)}}\quad \forall \quad {p}_{max}^{(n)}\ne 1\quad \wedge \quad {p}_{max}^{(n)}\ne {p}_{min}^{(n)}$$

28 $${p}_{min}^{(n+1)}={p}_{min}^{(n)}+({p}_{max}^{(n)}-{p}_{min}^{(n)})\frac{{A}_{set}^{(n)}-{A}_{drive}^{(n)}}{n\cdot {A}_{drive}^{(n)}}\quad \forall \quad {p}_{max}^{(n)}=1\quad \wedge \quad {p}_{max}^{(n)}\ne {p}_{min}^{(n)}$$

With this conditioning, the algorithm first tunes *p*_*max*_ and then *p*_*min*_ once the range of possible values is reached for *p*_*max*_. The upper range of *p*_*max*_ here is 1 and the lower range the actual value of *p*_*min*_ in each iteration.

### Sampled traffic activity and parking densities

The sampled traffic activity and parking density of cars in each zone can be derived from the state transition entries. If ${T}_{v,ij,t}^{dur}$ and ${T}_{v,ij,t}^{dis}$ are zero, it means that a car v is parked in zone i at time step t. By summing the number of parked cars in each time step and each zone, we can derive the spatio-temporal distribution parking and driving densities. For calculating the circadian rhythm of overall traffic activity, we sum the number of driving cars among all city zones for each time step separately. For all i = 1…N and t = 1…T, we introduce:

- *P*_*i*,*t*_: = the sum of sampled cars parking in zone i during time step t
- *D*_*i*,*t*_: = the sum of sampled cars driving in zone i during time step t
- $T{r}_{t}=\mathop{\sum }\limits_{i=1}^{N}\,{D}_{i,t}$ := the overall traffic activity at time step t

### Measured traffic activity and parking densities

The generated results can be validated for cities in which vehicle counts on street segments and parking measurements are available for the same period of time as the travel time data that we use for sampling. The city of Melbourne provides such data. Vehicle count sensors and between 5,073–9,288 underground parking sensors provide measurements of traffic activity between 2014–2017 and on-street parking bay occupancy with one second accuracy for the years 2011–2017^20^ Each vehicle count sensor measures the number of vehicles that passes the respective street segment and distinguishes between 22 types of different vehicles. Each parking sensor measures the arrival and departure times of cars being parked in their respective bays. A separate set of files provides information on latitudinal and longitudinal coordinates of the parking bays that are equipped with sensors. We use the mutual information of these files to first allocate the sensors and then to assign each sensor to one distinct city zone. Sensors are assigned to the city zone to which they have the shortest beeline distance. The parking density of each zone in each time step is then calculated as the share of time in which all its sensed parking bays are occupied by a car. With M being the subset of city zones for which parking measurements are available we introduce for all *m* ∈ *M* and t = 1…T:

- ${Y}_{m,t}^{Park}$: = the sum of parking bay occupancy measured in zone m during time step t
- ${Y}_{t}^{Traf}$: = the sum of vehicles counted on all sensed street segments during time step t

### Validation metric

We validate our model by calculating the Mean Squared Error between sampled and measured traffic activities and parking densities. We scale all sampled and measured values with respect to their minimum and maximum values among all time steps t = 1…T in order to be able to make sensible comparisons. For all validatable zones *m* ∈ *M* and t = 1…T, we calculate the sampled parking densities ${\widehat{P}}_{m,t}$, sampled traffic activities $\widehat{T}{r}_{t}$, measured parking densities ${\hat{Y}}_{m,t}^{Park}$ and measured traffic activities ${\hat{Y}}_{t}^{Traf}$ as:

29 $${\widehat{P}}_{m,t}=\frac{{P}_{m,t}-mi{n}_{t}\{{P}_{m,t}\}}{ma{x}_{t}\{{P}_{m,t}\}-mi{n}_{t}\{{P}_{m,t}\}}$$

30 $$\widehat{T}{r}_{t}=\frac{{\sum }_{i=1}^{N}\,{D}_{i,t}-mi{n}_{t}\{{\sum }_{i=1}^{N}\,{D}_{i,t}\}}{ma{x}_{t}\{{\sum }_{i=1}^{N}\,{D}_{i,t}\}-mi{n}_{t}\{{\sum }_{i=1}^{N}\,{D}_{i,t}\}}$$

31 $${\hat{Y}}_{m,t}^{Park}=\frac{{Y}_{m,t}^{Park}-mi{n}_{t}\{{Y}_{m,t}^{Park}\}}{ma{x}_{t}\{{Y}_{m,t}^{Park}\}-mi{n}_{t}\{{Y}_{m,t}^{Park}\}}$$

32 $${\hat{Y}}_{t}^{Traf}=\frac{{Y}_{t}^{Traf}-mi{n}_{t}\{{Y}_{t}^{Traf}\}}{ma{x}_{t}\{{Y}_{t}^{Traf}\}-mi{n}_{t}\{{Y}_{t}^{Traf}\}}$$

The percentual fits ${F}_{m}^{Park}$ and *F*^*Traf*^ between the measured and sampled values are then calculated as:

33 $${F}_{m}^{Park}=100 \% \cdot \left(1-\frac{1}{T}\mathop{\sum }\limits_{t=1}^{T}\,{({\widehat{P}}_{m,t}-{\hat{Y}}_{m,t}^{Park})}^{2}\right)$$

34 $${F}^{Traf}=100 \% \cdot \left(1-\frac{1}{T}\mathop{\sum }\limits_{t=1}^{T}\,{(\widehat{T}{r}_{t}-{\hat{Y}}_{t}^{Traf})}^{2}\right)$$

The average, minimum and maximum percentual fits of parking density for each sampled dataset can then be calculated as their values among all validated city zones *m* ∈ *M*, with |*M*| being the number of elements of set M, as:

35 $$\frac{1}{| M| }\sum _{m\in M}\,{F}_{m}$$

36 $$mi{n}_{m}\{{F}_{m}\}$$

37 $$ma{x}_{m}\{{F}_{m}\}$$

## Usage Notes

The performance of the presented model depends on the sparsity of the travel time data. The travel time data that is collected by Uber and that we have processed in this analysis contains different degrees of sparsity for each city and each dataset (Tables 3 & 4). We recommend users to evaluate the visualizations of the generated parking densities that we provide in a Graphics Interchange Format (GIF)^19^. We further sample parking densities with the same parameters for all datasets. The state transition properties that result from these samples are provided in the sampling_parameters.csv for each city. Users can increase the accuracy of the individual parking density maps that we provide by performing the additional computational work that is involved with the model selection that we introduce in the Jupyter Notebook instructions^18^.

## Data Availability

The travel time data that we use for sampling city-scale parking maps can be downloaded from the Uber Movement project website^17^. The car parking density maps and traffic activity rhythms that we derive from these datasets can be accessed on Harvard Dataverse^19^. Any data that is used for the validation of parking densities in the city of Melbourne is available on the website of the city of Melbourne^20^. The data that we generate for each sampled city consists of four different types of files: a first set of files starts with “results_parkingdensities” and contains the share of parked cars in each city zone (rows) during each hour of the day (columns); a second set of files starts with “results_trafficactivity” and contains the circadian rhythm of overall traffic activity; a third file is called “sampling_parameters.csv” and contains the chosen and resulting parameters of the model; the fourth and last file is called “zoneID_coordinates.csv” and contains the representative latitudinal and longitudinal coordinates of each city zone polygon.

## Code Availability

In addition to the description of our method here, we also provide code and instructions for reproducing the presented results and extending the developed model in Julia programming language^18^. In a first set of files we provide the contiguous programs that we use to generate the presented results and their validation; these are the files that end with “.jl”. They can be used to reproduce the generated results. In a second set of files that are written as Jupyter Notebook instruction we provide step by step explanations on how our model and the validation metric work. These files can be used to customize our method for individual modeling purposes and to better understand the modeling and validation steps; they end with “.ipynb”. The presented results are generated without indiviually performed model selections; they are produced with model parameters of *e*_*drive*_ = 0.5, *e*_*dest*_ = 2, *p*_*min*_ = 0.1 and *p*_*max*_ = 0.9 that are found to be good parameters for the validated cities. The results are further sampled with a total vehicle fleet size of 1,000 cars per city zone; this allows us to exploit the law of large numbers and converge towards realistic distributions of cars while keeping the calculation within the range of feasible computational time with moderate computational power.
